# Nephrogenic diabetes insipidus in initial stage of acute lymphoblastic leukemia and relapse after haploidentical hematopoietic stem-cell transplantation: A case report

**DOI:** 10.1097/MD.0000000000011157

**Published:** 2018-06-15

**Authors:** Dezhi Li, Qian Liu, Zhifang Feng, Qi Zhang, Saran Feng

**Affiliations:** a Department of Respiratory Medicine, Shandong Provincial Hospital Affiliated to Shandong University; b Department of Hematology, Shandong Provincial Qianfoshan Hospital, Jinan City; c Department of Endocrinology, Zhucheng Traditional Chinese Medicine Hospital, Zhucheng City, China; d Drug Discovery and Biomedical Sciences, College of Pharmacy, University of South Carolina, Columbia, SC.

**Keywords:** acute lymphoblastic leukemia, haploidentical stem-cell transplantation, nephrogenic diabetes insipidus, relapse, renal infiltration

## Abstract

**Rationale:**

Nephrogenic diabetes insipidus (NDI) rarely presents in the initial stage of acute lymphoblastic leukemia (ALL) and relapse due to renal infiltration is also rare.

**Patient concerns:**

A 19-year-old man presented with weakness, polydipsia, and polyuria for 1 month.

**Diagnoses:**

NDI was diagnosed with insignificant response to a water deprivation test after stimulation with vasopressin injection. Bone marrow examination combined with immunophenotypic analysis, cerebrospinal cytology, and abdominal ultrasonography confirmed the diagnoses of precursor B cell ALL with renal infiltration.

**Interventions:**

The patient accepted standardized combination chemotherapy and ultimately had sustained remission, and his polydipsia and polyuria disappeared after 3 days of treatment. The ALL relapsed 1 year later and he received haploidentical stem cell transplantation (haplo-SCT) from his father.

**Outcomes:**

One year later, he again developed NDI, with bilateral renal enlargement because of extramedullary relapse, leading to subsequent death.

**Lessons:**

This case demonstrates unusual early renal involvement in ALL presenting with initial NDI. Interestingly, the NDI returned with the relapse of renal infiltration 1 year after haplo-SCT. This case suggests that NDI was probably secondary to renal leukemic infiltration.

## 1 Introduction

Extramedullary infiltration is one of the known manifestations of acute lymphoblastic leukemia (ALL) and is associated with a very poor prognosis.^[1]^ In general, the sites of extramedullary involvement are mainly the central nervous system and testis.^[2,3]^ It is a relatively common finding in all age groups with renal involvement in ALL and develops as the disease progresses.^[4–6]^ However, infiltration in the early stages of ALL is rare.^[7,8]^ Early presentation of ALL as nephrogenic diabetes insipidus (NDI) with renal tubular acidosis is also rare. We report a patient who presented with NDI as the primary manifestation of ALL and died of a relapse of NDI with bilateral kidney infiltration 1 year after haploidentical stem-cell transplantation (haplo-SCT).

## 2 Case report

### 2.1 Ethical review and consent

This is not a clinical study. Therefore, ethic approval is not needed. Father of the patient provided the written informed consent.

### 2.2 Clinical course

A 19-year-old man presented with polydipsia and polyuria with muscle weakness for more than 1 month. His urine output was approximately 4 to 7 L/d. He later developed vomiting and paralysis. He was admitted to a local hospital and was hospitalized with severe hypokalemia (1.9 mmol/L) and metabolic acidosis (pH 7.22, pCO_2_ 26.0 mm Hg, HCO_3_ 10.6 mmol/L, lactate 7.1 mmol/L, base excess −15.6 mmol/L). The patient recovered from paralysis on the third hospital day with serum potassium level 3.1 mmol/L and normal blood gas results after appropriate therapy. He presented to our hospital for further evaluation. Physical examination was only significant for pallor. He did not have a family or personal history of neuromuscular, thyroid, or autoimmune disease.

### 2.3 Laboratory tests and diagnosis

At the time of admission, laboratory evaluation revealed the following abnormalities: white blood count 3.16 × 10^9^/L, hemoglobin 74 g/L, and platelet count 128 × 10^9^/L. Serum chemistry showed: sodium 140 mmol/L, chloride 108 mmol/L, potassium 3.18 mmol/L, calcium 1.89 mmol/L, creatinine 57.8 mmol/L, and bicarbonate 19 mmol/L. Urinary pH was 6.5, with sodium 80 mmol/L and potassium 18.68 mmol/L; chloride, calcium, and creatinine were normal. A 24-hour urine test started on the day of admission revealed the following: sodium 320 mmol/24 h, potassium 74.7 mmol/24 h, calcium 12.24 mmol/24 h, and protein 988.00 mg/24 h. Serum and urinary osmolality were 303 and 158 mOsm/kg, respectively. Growth hormone, insulin-like growth factor 1, thyroid hormone, adrenocorticotropic hormone, plasma aldosterone, renin activity, antinuclear antibody, and anti-Smith antibody were within reference range. Other chemistries and coagulation parameters were in normal range.

**Figure 1:**
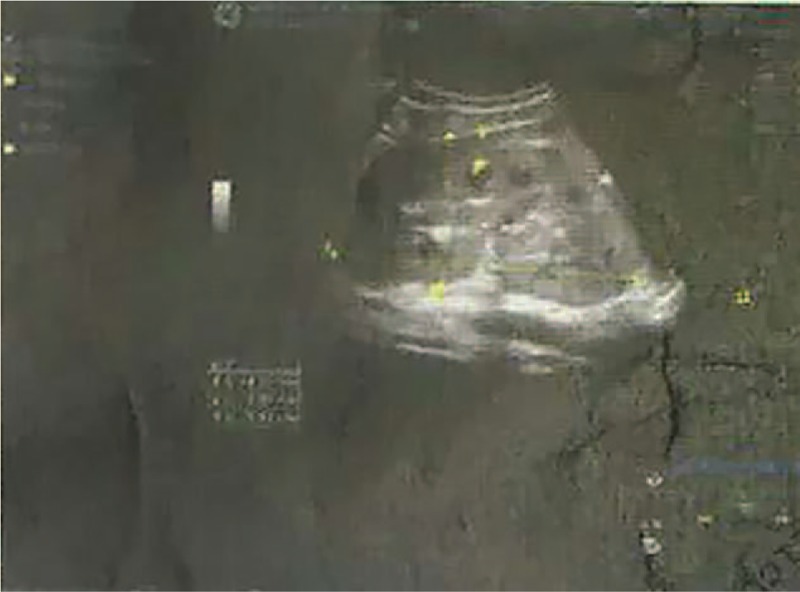
Abdominal ultrasonography revealed symmetrical homogenous kidneys with hyperechogenic pattern and poor corticomedullary differentiation.

Brain magnetic resonance imaging showed an abnormal pituitary gland, and he had an insignificant response to a water deprivation test after vasopressin injection. The findings suggested NDI. With overt acidosis (plasma bicarbonate <20 mmol/L) and urinary pH > 6.0, we confirmed the coexistence of renal tubular acidosis. Abdominal ultrasonography revealed symmetrical homogeneous kidneys with hyperechogenic pattern and poor corticomedullary differentiation (Fig. 1). Because of the hematologic abnormalities, bone marrow aspiration was performed and revealed 39% blast cells. The immunophenotypic analysis showed blast cells positive for CD34+, CD38+, CD10+, CD19+, CD22+, cyCD22+, CD79+, HLA-DR+, and TdT+. Cytogenetics showed normal male karyotype. Cerebrospinal cytology was negative. He was diagnosed with precursor B-cell ALL with acquired NDI. Leukemic infiltration of the kidneys may have led to polydipsia and polyuria.

### 2.4 Treatment and prognosis

**Figure 2:**
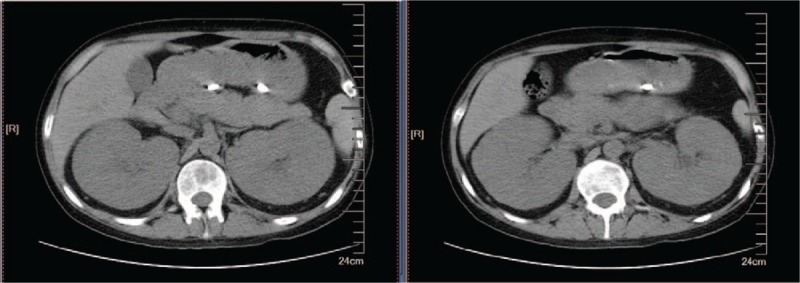
Abdominal computed tomography showing bilateral symmetrical, low-density, homogeneous renal enlargement.

This patient had an excellent response to standardized combination chemotherapy and achieved complete remission (CR) at the end of induction. His polydipsia and polyuria improved considerably after 3 days of chemotherapy. He refused the best therapy of allogeneic stem-cell transplantation (allo-HSCT) at that time. Unfortunately, leukemia relapsed after sustained remission for 1 year. As a human leukocyte antigen-matched related or appropriate unrelated donor was not available, haplo-SCT was an alternative. He received haplo-SCT from his father after a second CR. Unfortunately, 20% blast cells were found in bone marrow 6 months after haplo-SCT. Next, a graft-versus-leukemia effect was successfully induced with the intervention of donor lymphocyte infusion, which achieved sustained remission. At 1 year after haplo-SCT, the patient developed a fungal pulmonary infection and was found to have bilateral renal enlargement with low density on abdominal computed tomography (Fig. 2). Abdominal ultrasonography revealed symmetrical homogeneous kidneys with a hyperechogenic pattern and poor corticomedullary differentiation. The left kidney measured 17.1 cm × 9.6 cm, and the right kidney measured 16.9 cm × 9.7 cm. He returned 9 days later with muscle weakness, paralysis, polydipsia, and polyuria. Finally, he died of NDI and subsequent medullary relapse.

## 3 Discussion

Extramedullary infiltration of acute leukemia includes a wide variety of clinical manifestations and organ involvement, which may be present at initial diagnosis or relapse. Impairment of renal function and palpable nephromegaly are frequent initial manifestations as a result of renal infiltration in ALL.^[9]^ However, NDI is an unusual form of presentation in ALL with bilateral renal involvement.

The NDI is a hereditary or acquired condition caused by an improper response of the kidney to arginine-vasopressin, a nonapeptide hormone also known as antidiuretic hormone. This leads to a decreased ability to concentrate urine and is characterized by polydipsia, polyuria, and hyposthenuria in the presence of plasma hyperosmolality.^[10]^ NDI is most commonly secondary to kidney or other illness such as urinary tract obstruction, polycystic kidney disease, chronic infection, amyloidosis, sickle cell disease, granulomatous diseases, chronic infection, primary Sjögren syndrome, or various drugs that interfere with the action of vasopressin in the renal tubules.^[9,11]^ Unlike congenital NDI, acquired NDI is often reversible with correction of the causative problem.

In the present case, diabetes insipidus was the initial clinical presentation with renal tubular acidosis. Central diabetes insipidus, diabetes mellitus, and psychogenic polydipsia were all excluded by laboratory examination and administration of desmopressin. The diagnosis of NDI with renal tubular acidosis was made. The bone marrow aspirate, immunophenotypic analysis, and cytogenetic studies confirmed the diagnosis of early B-cell leukemia. Abdominal ultrasonography revealed symmetrical homogeneous kidneys with hyperechogenic pattern and poor corticomedullary differentiation. We concluded that the NDI with renal tubular acidosis was a secondary disease induced by renal infiltration with leukemic cells. Biopsy still serves a vital role in the diagnosis of extramedullary infiltration in ALL, which can define the precise nature of the infiltrate and guide therapy. However, biopsy may not favor because of thrombocytopenia, bleeding, or pain. In this case, remission chemotherapy was initiated immediately after diagnosis, and NDI symptoms rapidly corrected, also confirming renal involvement.

The optimal treatment for this case of precursor B-cell ALL with renal infiltration was allo-HSCT at the time of his first CR (CR1), but he refused. He successfully received haplo-SCT from his father after CR2. Unfortunately, he developed a second medullary relapse during follow-up, which conferred a poor prognosis. Donor lymphocyte infusion only provided sustained remission for 6 months. Surprisingly, he developed bilateral renal enlargement, and the NDI returned with polyuria and polydipsia with extramedullary leukemic relapse 1 year after haplo-SCT. To our knowledge, no similar case has been reported.

Unlike extramedullary relapse of leukemia in the central nervous system and testicles, which are “sanctuary sites” that protect leukemic cells from chemotherapy because of the blood-brain barrier and blood-testicular barrier, the phenomenon of NDI in leukemic renal infiltration and relapse was difficult to explain. Aquaporins, especially aquaporin-2 (APQ2), have a critical role in water regulation in the kidney.^[9]^ A reduction of APQ2 or interference with its transport to the cell membrane plays an essential role in the development of polyuria associated with many of the acquired forms of NDI.^[9,12,13]^ We hypothesized that in the process of infiltration, the secretion of matrix metalloproteinase by leukemic cells might injure or affect APQ2 expression, leading to increased water permeability of the cell membrane. Hypercalciuria, hypokalemia, and renal tubular acidosis are associated with decreased expression of AQP2, and may also have been involved in the mechanism of NDI in this case.

In conclusion, our case highlights the rare presentation of ALL with NDI due to bilateral renal infiltration at the time of diagnosis, with relapse after haplo-SCT. These findings indicated that NDI was probably secondary to renal leukemic infiltration. The molecular and biological mechanisms of NDI secondary to extramedullary renal infiltration in ALL require further investigation.

## Author contributions

**Data curation:** Zhifang Feng.

**Funding acquisition:** Saran Feng.

**Investigation:** Qian Liu, Zhifang Feng.

**Methodology:** Dezhi Li.

**Resources:** Qian Liu.

**Supervision:** Saran Feng.

**Writing – original draft:** Dezhi Li.

**Writing – review & editing:** Qi Zhang, Saran Feng.
